# Current trends, applications, and challenges in three-dimensional bioprinting for cardiovascular disease models and therapies

**DOI:** 10.1016/j.isci.2026.115519

**Published:** 2026-03-30

**Authors:** Bo Wang, Jing Zhang, Jipin Jiang, Chen Cheng, Yuanyuan Zhao

**Affiliations:** 1 Department of Gastroenterology, Tongji Hospital, Tongji Medical College, Huazhong University of Science and Technology, Wuhan, China; 2 Department of Oncology, Tongji Hospital, Tongji Medical College, Huazhong University of Science and Technology, Wuhan 430030, China; 3 Institute of Organ Transplantation, Tongji Hospital, Tongji Medical College, Huazhong University of Science and Technology, Key Laboratory of Organ Transplantation of Ministry of Education, National Health Commission and Chinese Academy of Medical Sciences, Wuhan, China; 4 Ultrasound Diagnosis Department, Maternal and Child Health Hospital of Hubei Province, Tongji Medical College, Huazhong University of Science and Technology, Wuhan, China

**Keywords:** Cardiovascular medicine, Bioengineering, Tissue engineering, Biotechnology, Biomaterials

## Abstract

Cardiovascular diseases (CVDs) remain the leading cause of death worldwide. Although conventional treatments such as surgery and transplantation have improved patient survival, they are subject to major limitations. Three-dimensional (3D) bioprinting presents new opportunities for treating CVDs, with the long-term objective of fabricating functional tissues that faithfully replicate native cardiac structure and key physiological functions, including contractile force, electrical conduction, and mechanical integrity. This review outlines the use of 3D bioprinting in modeling principal cardiac disorders, such as arrhythmias, structural heart defects, myocardial infarction, cardiac fibrosis, and heart failure. It further assesses the utility of bioprinting in developing disease models and advancing clinical therapies for these conditions. Finally, we address the ongoing challenges in implementing bioprinting and cardiac tissue engineering for CVDs and suggest possible avenues for improvement. Future studies should prioritize clinical translation and long-term follow-up to evaluate the durability and viability of bioprinted cardiac tissues.

## Graphical abstract

**Figure:**
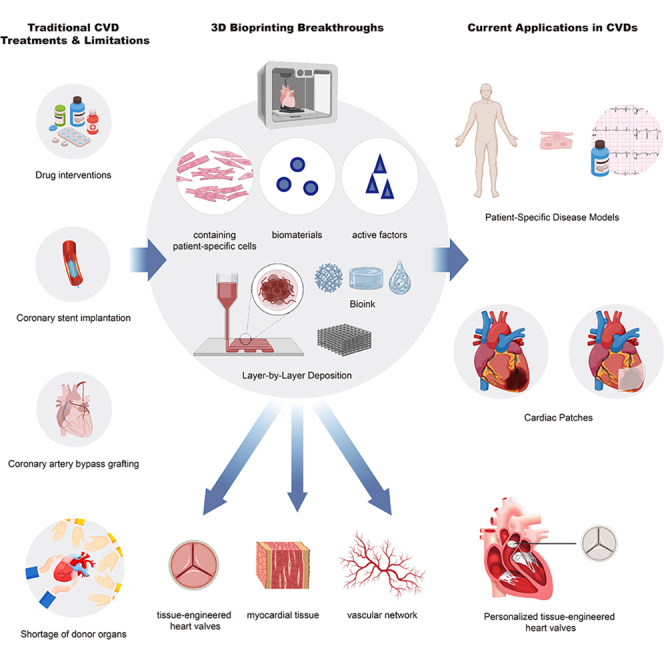

## Introduction

Cardiovascular diseases (CVDs) remain the foremost cause of death globally. According to the World Health Organization, CVDs are responsible for approximately 17.9 million fatalities annually, representing 32% of all deaths worldwide.^1^ The global burden of CVDs is projected to increase in the coming decades, largely due to an aging population.^2^ Conventional therapies, including pharmacotherapy, coronary stent placement, coronary artery bypass grafting, and organ transplantation, have saved numerous lives. However, these approaches are hampered by substantial limitations. A severe shortage of donor organs leaves many transplant candidates without viable options. Existing platforms for drug screening often inadequately replicate the intricate physiological environment of the human body, contributing to prolonged development timelines and high rates of failure. Furthermore, considerable inter-individual physiological variation frequently diminishes the effectiveness of standardized treatment protocols, hindering the achievement of truly personalized medicine.

Three-dimensional (3D) bioprinting, a major innovation within 3D printing technology, presents novel opportunities for addressing CVDs. This interdisciplinary field merges advanced manufacturing, materials science, and life sciences. It utilizes “bioinks,” composites of living cells, biomaterials, and bioactive factors, to construct functional living architectures via computer-guided, layer-by-layer deposition. The technology can accurately reproduce the complex curvature of heart valves, the anisotropic structure of myocardial tissue, and the elaborate branching networks of coronary arteries. Moreover, it allows for the precise 3D spatial organization of diverse cell types (e.g., cardiomyocytes, endothelial cells, fibroblasts, etc.) to mimic the native cellular microenvironment. The overarching objective is to engineer bioactive cardiovascular tissues that not only closely resemble natural tissues in morphology but also exhibit essential physiological functions such as contractility, electrical coupling, and mechanical strength.

**Figure 1:**
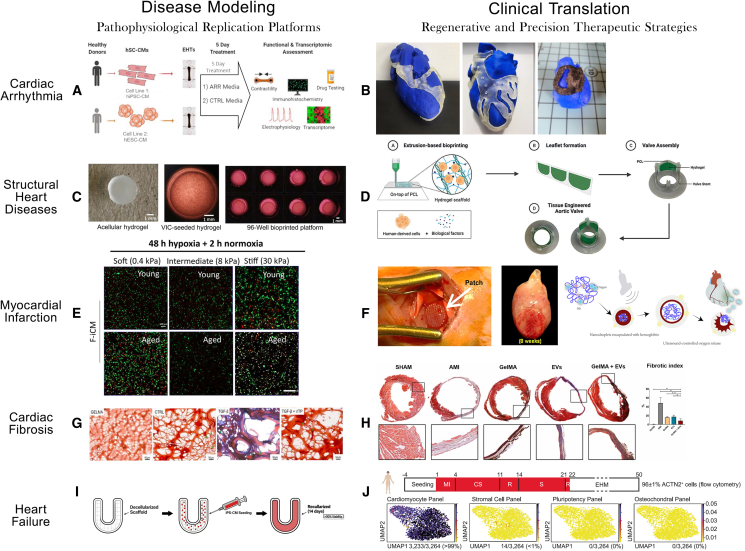
Applications of 3D bioprinting in cardiovascular disease modeling and clinical treatment (A) hiPSC-CM models simulate arrhythmogenic ion channel dysfunction and conduction block.^22^ (B) Patient-specific 3D-printed cardiac ablation guides aim to improve surgical accuracy, with various designs shown in MED625FLX resin.^23^ (C) A high-throughput bioprinted aortic valve array models the calcific aortic valve disease microenvironment for drug screening.^24^ (D) A trilayered tissue-engineered aortic valve scaffold is fabricated from a GelMA/PEGDA hydrogel reinforced with PCL fibers.^25^ (E) A bioprinted aged MI tissue model mimics the ischemic microenvironment and age-specific drug responses.^8^ (F) Preclinical studies show vascularized cardiac patches improve cardiac function in a porcine model and a strategy uses ultrasound-triggered oxygen release to enhance survival.^26^^,^^27^ (G) A tunable-stiffness bioprinted hydrogel platform studies myofibroblast differentiation and ECM deposition in fibrosis.^28^ (H) Injectable exosome-loaded hydrogel composites reduce fibrosis in a murine model.^29^ (I) Functional human myocardial tissue was engineered by reseeding hiPSC-CMs into decellularized heart scaffolds. (J) The illustration depicts the BioVAT-HF clinical trial, involving the first implantation of an iPSC-based heart patch in end-stage heart failure patients.^30^ Reproduced with permission from previous studies.^8^^,^^22^^,^^23^^,^^24^^,^^25^^,^^26^^,^^27^^,^^28^^,^^29^^,^^30^

In recent years, 3D bioprinting has achieved considerable progress in fabricating cardiovascular constructs and fostering tissue regeneration. Nevertheless, significant obstacles remain for its application in cardiac tissue engineering and CVD therapy. These include the development of functional, scalable vascular networks; managing immunogenicity; and ensuring the electromechanical maturity of engineered tissues. In this review, we outline the epidemiology and pathogenesis of major cardiac disorders, including arrhythmias, structural heart diseases, myocardial infarction (MI), cardiac fibrosis, and heart failure (Table 1). We subsequently examine the contributions of bioprinting to modeling these diseases and advancing their clinical management (Figure 1). Finally, we discuss enduring challenges in applying bioprinting and cardiac tissue engineering to CVDs and suggest potential avenues for resolution. Future investigations should emphasize clinical trials with extended follow-up to validate the long-term survival, integration, and durability of bioprinted cardiac tissues.

**Table 1:** The characteristics of epidemiology and pathogenesis of main cardiovascular diseases including cardiac arrhythmia, structural heart diseases, myocardial infarction, cardiac fibrosis, and heart failure

Disease	Epidemiology	Pathogenesis	Key risk factors
Cardiac arrhythmia	Global population ≥60 years: 1 billion (2019) Projected to double by 2050^3^	Ion channel dysfunction Abnormal electrical conduction Fibrotic tissue disruption^4^	Genetic mutations Myocardial ischemia Structural remodeling^4^
Structural heart diseases	5%–10% (65–74years) and 10%–20% (>75years) 23% of tertiary-care patients 40% with concurrent CAD^5^	-Degenerative: Leaflet calcification (aortic stenosis) Chordal rupture/myocardial infiltration (mitral valve) - Infective: Rheumatic heart disease/endocarditis - Congenital: Bicuspid aortic valve - Ventricular dysfunction: Papillary muscle displacement Annular dilatation (atrial fibrillation) - Ischemic: Post-MI remodeling (inferobasal MI → mitral regurgitation)^6^	Age (primary degenerative disease) Genetic mutations (e.g., bicuspid aortic valve) Infections: Rheumatic fever Infective endocarditis Heart failure (reduced ejection fraction) Atrial fibrillation (annular dilatation) Pulmonary hypertension (tricuspid regurgitation)^5^^,^^6^
Myocardial infarction (MI)	Global prevalence: 3.8% (<60years) → 9.5% (≥60years)^7^ Leading cause of CVD deaths	- Prolonged myocardial ischemia → cell death - Pathological progression: · Early changes (10–15 min): - Reduced glycogen - Sarcolemmal disruption - Mitochondrial abnormalities · Necrosis progression: - Subendocardium → subepicardium (hours) - Biomarker elevation^8^	Age Atherosclerosis Hypertension Dyslipidemia Diabetes mellitus Smoking Family history of CVD^7^^,^^9^
Cardiac fibrosis	Contributes significantly to CVD mortality (leading global cause of death) Age-dependent progression^10^	TGF-β/Smad pathway activation Myofibroblast differentiation Excessive ECM deposition^11^^,^^12^	Obesity Diabetes alcohol consumption post-viral dilated cardiomyopathy hypertrophic cardiomyopathy^13^^,^^14^^,^^15^^,^^16^
Heart failure (HF)	Incidence: 1.0–4.6/1,000 person-years (varies by ethnicity)^12^^,^^17^^,^^18^ High mortality^19^	Neurohormonal activation (RAAS) Pathological hypertrophy Ventricular remodeling^20^	MI history Diabetes Hypertension Valvular diseases^21^

## Cardiac arrhythmia

### Epidemiology and pathogenesis of cardiac arrhythmia

Cardiac arrhythmias impose a considerable global health burden. According to the World Health Organization, the worldwide population aged 60 years and older reached 1 billion in 2019 and is expected to double by 2050.^3^ The pathogenesis of arrhythmias involves abnormalities in cardiac electrical activity, encompassing ion channel dysfunction, impaired conduction between cardiomyocytes, and tissue heterogeneity, all contributing to rhythm disturbances.^31^^,^^32^^,^^33^ These mechanisms are frequently initiated by genetic mutations, myocardial ischemia, or structural remodeling.^4^ Existing therapeutic approaches, including pharmacotherapy and catheter ablation, face limitations such as adverse side effects, high recurrence rates, and incomplete restoration of cardiac function.

## Bioprinting in the establishment of cardiac arrhythmia models and clinical treatments

### *In vitro* disease modeling

3D bioprinting supports the development of physiologically relevant arrhythmia models through precise, layer-by-layer deposition of cells, biomaterials, and growth factors. Considerable progress has been achieved in creating humanized arrhythmia models. For example, Williams et al. used human induced pluripotent stem cell-derived cardiomyocytes (hiPSC-CMs) and cardiac fibroblasts to fabricate 3D microtissues that closely recapitulate the intricate electrophysiological features of arrhythmias. This model not only offers a robust platform for investigating arrhythmia mechanisms but also serves as a valuable tool for drug screening and therapy development.^22^ In addition, 3D-bioprinted cardiac organoids can mimic the structural and functional complexity of the heart, providing versatile systems for studying disease pathology and assessing treatment strategies, while bioprinted tissue engineering scaffolds present regenerative options for damaged myocardium.^34^^,^^35^

## Therapeutic strategies and clinical management

The high precision and capacity for personalization offered by 3D bioprinting hold transformative potential for the treatment of arrhythmias. A central innovation in this area involves the development of electrically conductive bioinks, which incorporate nanoscale fillers such as graphene, carbon nanotubes, metallic nanoparticles, and MXenes, as well as conjugated polymers like polyaniline, polypyrrole, and poly(3,4-ethylenedioxythiophene). These materials enable the fabrication of functional myocardial tissues capable of repairing damaged cardiac conduction systems and enhancing electrical synchronization.^36^^,^^37^ For example, Van Zyl et al. developed an injectable hydrogel composed of nanocellulose and carbon nanotubes that successfully restored electrical conduction across ablated tissue in canine hearts.^38^ In another study, a poly-3-amino-4-methoxybenzoic acid-gelatin construct significantly lowered the pacing threshold voltage in rat hearts, thereby extending pacemaker battery life.^39^ A notable advancement involves the use of polyethylene glycol-based hydrogels to convert coronary veins into conformable pacing elements. These constructs can stimulate the deep myocardium, synchronize myocardial activation, and achieve painless defibrillation by eliminating re-entrant circuits.^40^

3D bioprinting is also paving the way for next-generation bioelectronic therapies. Pong et al. reported a method for rapidly producing 3D-printed mapping devices derived from magnetic resonance imaging data. These devices, which incorporate flexible electronic arrays conforming to the epicardial contours of the atria and ventricles, facilitate high-resolution epicardial electrical mapping and ablation *in vivo*, as demonstrated in preclinical models and *ex vivo* human hearts.^41^

Furthermore, 3D bioprinting is advancing clinical arrhythmia management by enabling the creation of patient-specific anatomical models from preoperative imaging data (CT, MRI, etc.). These models enhance the precise visualization of arrhythmogenic substrates and support improved surgical planning. For instance, Candelari et al. developed 3D-printed surgical guides that notably increased the accuracy of ischemic scar ablation while reducing procedural complications.^42^ Studies have confirmed the feasibility of using 3D-printed materials such as MED625FLX resin and thermoplastic polyurethane elastomer (TPU95A) to fabricate cardiac ablation guides, establishing a safety threshold (thickness ≥1.0 mm) and supporting personalized, precision ablation strategies.^23^ In a randomized controlled trial involving patients with atrial fibrillation, the use of 3D-bioprinted left atrial models to assist in radiofrequency ablation significantly shortened procedural duration and reduced intraoperative radiation exposure.^43^ These developments allow clinicians to simulate complex procedures, such as pacemaker lead placement, thereby minimizing associated risks.^42^^,^^44^

## Structural heart diseases

### Epidemiology and pathogenesis of structural heart diseases

Structural heart diseases, a global health concern, encompass a range of conditions that affect the heart’s morphology and function, including valvular disorders, congenital defects (e.g., septal defects, aortic coarctation, etc,), cardiac tumors, and acquired structural abnormalities. Epidemiological data indicate that moderate or severe valvular heart disease affects 23% of patients in tertiary care, with 40% of these individuals also presenting with coronary artery disease. Its prevalence increases from 17% in individuals under 45 years to 28% in those over 75. Despite stable rates of MI and secondary prevention requirements in the United States over the past two decades, surgical risks lead to conservative management in nearly half of eligible patients.^5^ The pathogenesis of structural heart diseases involves complex gene-environment interactions throughout life. Conventional treatments typically rely on invasive surgical procedures and the implantation of artificial devices, which are associated with inherent risks.^6^ Current interventions for valvular diseases, such as mechanical and bioprosthetic valves, present limitations including thrombosis, calcification, finite durability, and an inability to adapt to patient growth. Similarly, traditional repairs for congenital defects often utilize synthetic patches or prostheses that lack growth potential and biological integration. Recent advances in 3D bioprinting, however, offer new avenues for personalized and regenerative therapeutic approaches.

3D bioprinting enables customized valve design across three principal domains: the fabrication of tissue-engineered valves, the prototyping of anatomical valve models, and the optimization of polymer-based heart valves.^45^

## Bioprinting in the establishment of structural heart diseases models and clinical treatments

### Applications in valvular heart disease

In heart valve engineering, both 3D printing and 3D bioprinting technologies find broad application. 3D printing techniques, such as stereolithography and fused deposition modeling, are employed to fabricate acellular valve prototypes for hemodynamic testing and surgical simulation. In contrast, 3D bioprinting methods, including direct ink writing and bioplotting, utilize cell-laden bioinks to create tissue-engineered heart valves (TEHVs) designed for regeneration and growth potential. Essential functional criteria for these engineered valves include optimal hydrodynamic performance, enhanced durability, excellent biocompatibility, and compatibility with transcatheter implantation techniques.^46^ The Cassandra team developed a 3D-bioprinted aortic valve array model using gelatin methacrylate (GelMA)/hyaluronic acid methacrylate (HAMA) hydrogels to replicate the biomechanics of the fibrous layer in calcific aortic valve disease (CAVD). This model recapitulates human CAVD tissue at the proteomic level with high fidelity (94% match versus 70% in conventional 2D models) and has elucidated the pivotal role of extracellular vesicles (EVs) in the calcification process, providing a high-throughput platform for drug screening and personalized therapeutic development.^24^ In another study, valvular interstitial cell (VIC) scaffolds were successfully 3D-bioprinted and maintained in culture for up to 28 days using a hydrogel composed of 2% alginate and 8% gelatin, with VICs sourced from sheep aortic valves. This work offers a novel tissue-engineered cellular model for investigating the mechanisms underlying calcific CAVD.^47^

Tissue engineering enables the design of patient-specific heart valves that replicate the native valve’s trilaminar structure, comprising the fibrosa, spongiosa, and ventricularis layers, by seeding scaffolds with a combination of endothelial cells, interstitial cells, and smooth muscle cells.^48^ This strategy provides favorable mechanical properties, supports cellular proliferation and valve regeneration, facilitates personalized therapy, and lowers the risk of immune rejection. For example, investigators have fabricated a 3D-bioprinted TEHV using a GelMA/poly(ethylene glycol) diacrylate (GelMA/PEGDA) hydrogel scaffold reinforced with polycaprolactone (PCL) to emulate the layered architecture of an aortic valve.^25^ The scaffold sustained cellular function and enhanced the expression of extracellular matrix (ECM) proteins, showing remodeling capacity under dynamic culture conditions and presenting a promising alternative for valve replacement.

The integration of 3D weaving and 3D bioprinting has markedly progressed cardiac valve tissue engineering. Methods such as shape weaving construct intricate valve geometries using yarns of varying lengths, while dedicated 3D weaving platforms generate detailed valve scaffolds. Specialized software converts mesh designs into weaving instructions, simplifying the production of durable, long-lasting valve prototypes.^49^ In tissue-engineered heart valve approaches, the *in vivo* TEHV strategy involves subcutaneously implanting a valve-shaped mold, which becomes enveloped by fibrous tissue for subsequent use in replacing diseased valves. The *in situ* TEHV strategy, in contrast, directly places a biodegradable scaffold at the site of the damaged valve to stimulate endogenous regeneration.

### Application in congenital heart disease

3D bioprinting technology is reshaping the treatment paradigm for congenital heart disease, with its utility extending from precise diagnosis to functional repair. In the field of disease modeling, this technology advances surgical planning from static visualization to dynamic simulation. For instance, Tomov et al. conducted hemodynamic analysis by fabricating a perfusable vascular model of tetralogy of Fallot. Meanwhile, *in vitro* models of congenital heart disease constructed using hiPSC-CMs and GelXA Laminink-521 bioink offer a valuable platform for investigating pathological mechanisms and screening potential therapeutics, although they remain at the preclinical research stage.^50^^,^^51^ Historically, 3D printing efforts related to ventricular septal defects have largely concentrated on producing physical anatomical models from patient CT or MRI data. These models are employed for preoperative planning, surgical simulation, such as training for defect closure, and medical education.^52^

A key application involves the repair of septal defects. Ye et al. advanced this field by combining 3D bioprinting technology with functionally specialized atrioventricular canal cardiomyocytes to create a novel cardiac patch. This engineered construct achieves both anatomical closure of the defect and restoration of physiological electrical conduction, effectively preventing postoperative conduction block.^53^

In the broader context of tissue repair, the technology has enabled a progression from simple “patches” to functional “living tissues.” Ong et al. generated spontaneously beating cardiac patches using scaffold-free bioprinting, while Noor et al. further produced personalized, vascularized patches from patient-derived decellularized ECM bioinks.^54^^,^^55^ Kang et al. utilized mesenchymal stromal cells obtained from patients with cyanotic congenital heart disease, which naturally exhibit hypoxia preconditioning, together with cytokine-functionalized collagen scaffolds.^56^ This strategic combination of cells and scaffold design yielded engineered patches that synergistically enhanced both angiogenesis and cell survival during right ventricular outflow tract repair.^56^ In the development of functional grafts, work by Duan’s team (2012–2014) confirmed the feasibility of fabricating cellularized bioprosthetic valves via bioprinting, whereas Kawai et al. demonstrated the translational potential of tubular cardiac tissues capable of synchronous contraction and vascularization as implants with growth potential.^57^^,^^58^ Lewis-Israeli et al. found that self-assembling human heart organoids intrinsically develop a resident macrophage population, thereby recapitulating the innate immune microenvironment of the developing heart and offering a novel model to study the role of immune cells in cardiac development and inflammatory cardiomyopathies.^59^ Together, these advances are steering the treatment of congenital heart disease toward greater personalization, functionalization, and regenerative capacity.^60^

## Biomaterials for cardiac bioprinting

Various biomaterials serve distinct purposes within this technology. Fibrin, characterized by rapid polymerization and tunable degradation, is employed to fabricate biodegradable scaffolds. Collagen, valued for its inherent cell-binding properties, yields scaffolds with favorable biocompatibility and mechanical performance. Polysaccharides, benefiting from biodegradability and biocompatibility, are used to produce scaffolds that support cell adhesion and growth. Hyaluronic acid hydrogels present printing challenges due to high viscosity, though their printability can be improved through chemical modification. PEG hydrogels can enhance cell adhesion and proliferation when combined with other scaffold materials, but require solutions to challenges such as rapid degradation and cytotoxicity. PEGDA hydrogels, as a more durable photocurable alternative, have demonstrated good cytocompatibility and support for cell viability in both *in vitro* and *in vivo* evaluations.^35^^,^^61^ Compared to existing prosthetic heart valves, biodegradable polymer valves offer advantages including the elimination of donor organ requirements and lifelong anticoagulation therapy, as well as a reduced risk of infection. ECM components can be used to modify the surface of polymers such as poly(styrene-block-isobutylene-block-styrene), improving hydrophilicity, reducing protein and platelet adsorption, lowering thrombosis risk, and promoting endothelial cell adhesion and proliferation. These characteristics position ECM-modified materials as a promising option for fabricating polymer heart valves.^62^

## Myocardial infarction

### Epidemiology and pathogenesis of MI

CVDs remain the foremost cause of death worldwide, representing a major public health challenge due to their high incidence, mortality, and unfavorable prognosis. A synthesis of 42 studies encompassing 34.9 million individuals reports a global MI prevalence of 3.8% among persons under 60 years of age, rising sharply to 9.5% in populations aged 60 years or older, reflecting a marked age-dependent increase in disease burden.^9^ MI is fundamentally defined by elevated cardiac biomarkers in conjunction with clinical evidence of myocardial ischemia, such as symptoms, electrocardiographic changes, imaging findings, or angiographic evidence, and is classified into five clinical types.^7^ MI results from prolonged myocardial ischemia leading to cardiomyocyte death. Early ultrastructural alterations, including depleted cellular glycogen, relaxed myofibrils, sarcolemmal disruption, and mitochondrial abnormalities, can be observed within 10–15 min after the onset of ischemia.^7^ Necrosis typically extends from the subendocardium toward the subepicardium over subsequent hours. Cardiac troponin I and T are the preferred biomarkers for detecting myocardial injury; levels exceeding the 99th percentile upper reference limit signify injury, which may be acute or chronic.

## Bioprinting in the establishment of mi models and clinical treatments

### *In vitro* disease modeling

Recent progress in 3D bioprinting has demonstrated considerable potential for reconstructing infarcted myocardium. A 2019 study reported the first bioprinted aged cardiac tissue model using hiPSC-derived cardiomyocytes. By employing stiffness-mimicking scaffolds that replicate the mechanical properties of the aging heart and inducing accelerated cellular senescence, the engineered tissues recapitulated age-associated functional decline, pharmacological responses, and molecular pathology within four months.^8^ This bioprinted platform allows precise modeling of aging-related CVD mechanisms and therapeutic evaluation. Additionally, research has shown that combining hiPSC-derived cardiomyocytes with fibroblasts and embedding them in a collagen hydrogel can promote the self-assembly of cardiac tissue-like structures. These constructs not only partially preserve native cardiac tissue alignment but also generate contractile forces up to three times greater than those of spheroid-based tissue models.^63^ This method has substantially improved the contractile and electrical conduction properties of engineered cardiac tissues, presenting a promising new avenue for cardiac regeneration and disease modeling.

## Therapeutic strategies: Cardiac patches and injectable hydrogels

Stem cell-laden 3D-bioprinted cardiac patches represent a promising therapeutic strategy for ischemic cardiomyopathy. These patches incorporate diverse cell sources: multipotent stem cells such as mesenchymal stem cells (MSCs), valued for their paracrine and immunomodulatory effects but limited cardiomyogenic potential; and pluripotent stem cells such as induced pluripotent stem cells (iPSCs), which can differentiate into functional cardiomyocytes but require careful management of tumorigenicity risks. Additional cell types include embryonic stem cells (ESCs), possessing high plasticity but associated with ethical considerations, and cardiac progenitor cells, which are tissue-specific yet difficult to expand in culture.^64^ The objective of this approach is to counteract fibrosis, stimulate myocardial regeneration, and incorporate effective vascularization. For example, Lee et al. fabricated a GelMA-based patch seeded with iPSC-derived cardiomyocytes (iPSC-CMs). In rat models of MI, this patch improved left ventricular ejection fraction by 18% through enhanced contractility and reduced fibrotic remodeling.^64^^,^^65^

Notably, recent preclinical advances in 3D-bioprinted cardiac patches have shown considerable therapeutic potential for MI. Hwang et al. developed a vascularized patch delivering follistatin-like 1, which effectively promoted regeneration of ischemic tissue and restored cardiac function in rats.^66^ In parallel, Gil et al. engineered CT-visible patches by incorporating gold and gadolinium nanoparticles into GelMA bioinks, enabling non-invasive monitoring post-implantation while supporting tissue repair.^67^ Furthermore, Brazhkina et al. designed an innovative auxetic patch laden with iPSC-CMs that demonstrated synchronous expansion-contraction dynamics and promoted angiogenesis.^68^

Beyond patches, injectable hydrogels constitute another key therapeutic modality. An injectable GelMA hydrogel functionalized with EVs derived from human umbilical vein endothelial cells demonstrated efficacy in acute MI by attenuating fibrosis, improving cardiac contractility, and stimulating angiogenesis. Molecular profiling (miRNome/proteomics) confirmed the bioactive role of EVs, supporting a cell-free strategy that may avoid immune rejection and tumorigenic risks in regenerative therapy.^29^ Another innovative approach utilizes injectable hydrogels loaded with exosomes isolated from MSCs. These hydrogels reduced infarct size by 30% in murine models, mediated through modulation of inflammation and promotion of angiogenesis.^69^ In addition to synthetic hydrogels, decellularized ECM (dECM) scaffolds sourced from porcine cardiac tissue show promise by preserving native biochemical signals, thereby enhancing cell adhesion and differentiation. A 2021 study reported that dECM scaffolds attenuated inflammatory responses and improved systolic function in rabbit models.^69^^,^^70^

### Vascularization and electrophysiological integration

Vascularization represents a critical challenge in tissue engineering, particularly for ensuring the survival and functional integration of bioprinted cardiac constructs. One innovative strategy involves the co-printing of endothelial cells with sacrificial bioinks to generate perfusable microchannel networks. Zhang et al. demonstrated that such vascularized constructs enhanced capillary density by 40% in porcine hearts, which is essential for adequate oxygen delivery to implanted tissues.^65^ Additionally, the use of multicellular bioinks, combining cardiomyocytes, fibroblasts, and endothelial cells within fibrin-alginate hydrogels, has been shown to better replicate native tissue heterogeneity, thereby promoting electromechanical integration and improving overall tissue function.^69^ The incorporation of acoustic phase-transition nanodroplets and oxygen-loaded hemoglobin has further enabled non-invasive, spatiotemporally controlled oxygen delivery, addressing key limitations in engineered tissue viability and post-infarction regeneration.

Restoring electrical conduction in infarcted myocardium is equally crucial. Electroconductive materials, such as graphene oxide-enhanced hydrogels, have been developed to re-establish electrical propagation. A 2023 study reported synchronized contraction in bioprinted human cardiac tissues, achieving conduction velocities of 25 cm/s, a value comparable to that of native myocardium.^71^ In another 2023 investigation, an ultrasound-responsive nano-oxygen carrier was designed to achieve controlled oxygen release triggered by low-intensity pulsed ultrasound. This approach significantly improved the permeability of 3D-bioprinted GelMA-based cardiac patches and enhanced cellular survival. By mitigating hypoxia and preserving mitochondrial function, the strategy effectively promoted cardiac repair and revascularization in MI models.^27^

### Preclinical validation and translational outlook

Preclinical investigations and translational research have yielded important insights into the therapeutic potential of 3D bioprinting for MI. A 2024 study employing bioprinted engineered heart tissues in porcine MI models reported 60% functional recovery at six months post-implantation, accompanied by evidence of neuromuscular junction formation.^70^^,^^72^ This outcome highlights the capacity of bioprinted constructs to support substantial cardiac functional restoration. Furthermore, paracrine signaling from mesenchymal stem cell (MSC)-laden constructs has been shown to enhance endogenous repair processes through the secretion of factors such as VEGF and IGF-1, even in the absence of long-term MSC survival.^73^ The development of hybrid devices that integrate bioprinted tissues with wearable sensor technology has further advanced the field by enabling real-time monitoring of graft performance in large animal models such as sheep. This integrated approach helps to identify and address post-implantation complications, thereby improving the translational feasibility of bioprinted cardiac tissues.^70^

## Cardiac fibrosis

### Epidemiology and pathogenesis of cardiac fibrosis

Cardiac fibrosis constitutes a major public health challenge, closely associated with nearly all forms of CVD, the leading cause of death, morbidity, and disability worldwide.^10^^,^^74^ It primarily involves pathological myocardial remodeling, characterized by excessive deposition of ECM proteins within cardiac parenchymal tissue.^75^^,^^76^ In 2019, the Translational Research Committee of the Heart Failure Association of the European Society of Cardiology categorized cardiac fibrosis into “reparative/replacement fibrosis” and “reactive interstitial fibrosis.”^77^^,^^78^ Among these, “replacement fibrosis” substitutes necrotic cardiomyocytes with ECM and fibroblasts. “Reactive interstitial fibrosis,” in contrast, expands the interstitial and perivascular spaces without cardiomyocyte loss or fundamental alteration of myocardial bundle architecture, whereas “replacement fibrosis” disrupts the continuity of muscle bundles while preserving overall tissue integrity.^79^ Different types of fibrosis share several common features. For example, under systemic pressure overload, fibrosis is often diffuse and responsive to ongoing mechanical stress. Following acute injury such as MI, fibrosis initially serves a reparative role, leading to localized scar formation that replaces lost cardiomyocytes. Extensive cardiac fibrosis reduces tissue compliance and contributes to adverse outcomes, including cardiomyocyte apoptosis, hypertrophy, and eventual heart failure. Numerous factors have been identified as risk contributors for cardiac fibrosis, including Numerous factors have been identified as risk,^80^ diabetes,^13^ alcohol consumption,^14^ post-viral dilated cardiomyopathy,^15^ and hypertrophic cardiomyopathy^16^ among others.

Cardiac myocytes, unlike most human tissue cells which possess robust regenerative capacity, are highly susceptible to injury and rupture due to their limited ability to regenerate. To compensate for lost myocytes and protect the heart from further damage, fibrosis has evolved as an adaptive repair mechanism.^81^ In this process, fibroblasts play a central role. Upon injury or stress, fibroblasts become activated and differentiate into myofibroblasts. Subsequent apoptosis of these myofibroblasts can lead to rapid scar formation within damaged tissue. Multiple *in vivo* and *in vitro* studies have demonstrated that the TGF-β1/Smad signaling pathway is critically involved in regulating cell growth and differentiation, wound repair, and ECM production.^82^^,^^83^ Additionally, the Hippo signaling pathway has been identified as a novel pathway contributing to fibroblast activation.^84^ Collectively, persistent fibrotic responses can distort cardiac architecture, impair heart function, and further elevate the risk of progressive fibrosis. Ultimately, cardiac fibrosis induces structural alterations, reduces electrical coupling between myocytes, promoting focal electrical activity, and accelerates adverse cardiac remodeling.

## Bioprinting in the establishment of cardiac fibrosis models and clinical treatments

### *In vitro* disease modeling

Currently, most studies employ two-dimensional (2D) cell culture models stimulated with profibrotic factors such as TGF-β. This is because TGF-β1 signaling represents the principal pro-fibrotic pathway in cardiac fibrosis and may interact with the YAP-Hippo transcriptional pathway. An ideal cardiac fibrosis model should replicate the complex disease microenvironment, encompassing fibroblast activation and myofibroblast differentiation, excessive and mechanically altered ECM deposition, impaired electrical coupling and contractility, and inflammatory responses.^85^ Building on this, Ragazzini et al. developed a 3D model of cardiac ECM remodeling by fabricating tissue blocks with a tunable-stiffness GelMA bioink seeded with human cardiosphere-derived stromal cells.^28^ Compared with 2D models, 3D culture systems better represent the *in vivo* environment, enabling researchers to study the interactive mechanisms driving cardiac fibrosis onset and to evaluate drug effects.^86^ 3D cellular aggregates, such as spheroids and organoids, can serve as fibrosis models due to their ability to support cell-cell interactions. Spheroids typically incorporate cardiac fibroblasts, cardiomyocytes, and endothelial cells to account for vascularization.^87^ Such models support cellular physiological activity and ECM remodeling and can be used to assess therapeutic interventions. However, spheroids lack tissue-specific architecture and cellular organization^85^ Organoids, in contrast, can address some of these limitations through self-organization that mimics the structure and function of specific organs or tissues. Nevertheless, most cardiac organoids model healthy myocardium; artificial injuries (e.g., cryoinjury) or the introduction of risk factors are required to induce fibrotic phenotypes.^88^^,^^89^ Although 3D bioprinting has been widely adopted to replicate cardiac microenvironments, a bioprinted model that fully captures the hallmark pathological complexities of cardiac fibrosis, including progressive ECM dysregulation, disruption of cardiomyocyte architecture, and altered tissue mechanics, has not yet been established. For instance, Basara et al. bioprinted a model with three distinct regions mimicking the remote, border, and scar zones of aged human post-infarct myocardium by using different hydrogel compositions (including GelMA, methacrylated hyaluronic acid, collagen, and photoinitiators) for each region.^90^ This model may be applied in aged cardiac microenvironment studies and for testing novel therapeutics but does not constitute a comprehensive 3D-bioprinted cardiac fibrosis model. In fact, excessive ECM deposition and progressive architectural disruption of cardiomyocytes are defining features of cardiac fibrosis. Accordingly, various bioprinting techniques and materials, such as PCL-based scaffolds, have been used to create constructs that mimic the disorganized structure and increased stiffness of fibrotic tissue.^91^^,^^92^ Furthermore, dECM synthesized by cardiac fibroblasts can be modified to simulate fibrotic pathological conditions. Cardiac fibrosis models still face considerable gaps in the realm of personalized medicine. The use of human cardiac fibroblasts (hCFs) is constrained by high donor-to-donor variability and limited access to surgical samples. Differentiating hCFs from hiPSCs offers a viable alternative.^93^ Another potential strategy for personalized modeling involves isolating immune cells, such as monocytes, from patient blood samples.^94^

### Potential therapeutic applications

Therapeutic strategies targeting fibrosis are inherently anti-fibrotic in nature. The primary objective is not the delivery of new cardiomyocytes, but rather the halting or reversal of pathological remodeling. Bioprinted models serve predominantly as platforms for drug screening to identify compounds capable of inhibiting key pro-fibrotic pathways, such as those mediated by TGF-β and YAP-Hippo.^84^ Future research will consequently focus on developing and refining strategies to enhance the fidelity and utility of myocardial fibrosis models.

## Heart failure

### Epidemiology and pathogenesis of heart failure

Heart failure is defined by the American College of Cardiology/American Heart Association as a complex clinical syndrome resulting from any structural or functional cardiac impairment that compromises ventricular filling or ejection.^95^ Multiple clinical diagnostic criteria have been established, ranging from the Framingham criteria^96^ to the Cardiovascular Health Study criteria.^97^ The prevalence of heart failure is strongly age-dependent.^11^ Its incidence is further influenced by race, sex, diabetes, obesity, ethnicity, and socioeconomic factors. For instance, one study reported incidence rates per 1,000 person-years of 4.6 among African American, 3.5 among Hispanic American, 2.4 among White American, and 1.0 among Chinese American populations.^12^^,^^17^^,^^18^ Additional studies have identified income and educational attainment as factors associated with new-onset heart failure.^98^ Overall, heart failure carries a high mortality rate that surpasses that of many cancers.^19^

Numerous conditions can precipitate heart failure, including arrhythmias, MI/ischemia, diabetes, hypertension, and valvular heart disease. These disorders can progressively lead to cardiac hypertrophy, fibrosis, and ultimately heart failure.^20^ Central to this progression is pathological cardiac remodeling, which involves neurohormonal activation. Although current pharmacotherapies, such as β-blockers, angiotensin-converting enzyme inhibitors/angiotensin receptor blockers, and aldosterone antagonists, have demonstrated benefit in heart failure management, their therapeutic efficacy remains insufficient. Consequently, identifying novel pathological mechanisms to develop more effective treatments represents an urgent priority.

## Bioprinting in the establishment of heart failure models and clinical treatments

### Disease modeling

In recent years, stem cell therapy has shown promise for treating ischemic heart disease and heart failure. The delivery of patient-derived stem cells has been demonstrated to promote regeneration of damaged cardiac tissue.^21^^,^^99^ 3D bioprinting offers the advantage of incorporating patient-derived stem cells to create personalized tissue constructs, also referred to as 3D-bioprinted cardiac patches.^100^^,^^101^ This approach may ultimately provide an alternative to heart transplantation with comparable potential for cardiac regeneration. Stevens et al. first described a method to generate scaffold-free patches of human cardiac tissue using cardiomyocytes derived from ESCs; these patches exhibited synchronous calcium transients and spontaneous contractile activity.^102^ However, similar to the situation with cardiac fibrosis, a comprehensive 3D-bioprinted model of heart failure has not yet been established.

### Therapeutic strategies

The principal therapeutic focus for heart failure lies in developing functional cardiac patches for implantation. Adequate vascularization, remuscularization, and a native-like ECM remain critical for ensuring long-term cell survival and function *in vivo*.

Regarding vascularization and remuscularization, Jebran et al. fabricated cardiac patches that were heterotopically grafted into rat hearts following transmural aortic punch injury, observing partial remuscularization and vascularization of the transplanted tissue.^103^ In a study by Kawai et al., 3D-printed engineered heart tissue grafts implanted around the abdominal inferior vena cava of mice exhibited pulsatility and supported neovascularization.^58^ However, rodents are known to promote rapid vascularization of transplanted tissues, which may circumvent some challenges encountered in large animal models.^104^ In large animal models, Reinsch et al. created mesh-structured tissue patches for transplantation in pigs and demonstrated dose-dependent remuscularization and improved left ventricular function in a guinea pig injury model, though vascularization was not addressed.^105^ A further challenge for cardiac patches in large animals is establishing functional connections with host vasculature. Endo et al. developed a pre-vascularization technique that generates vascularized human myocardial tissue complete with a feeding artery and draining vein.^106^ However, this method has not yet been applied in cardiac transplantation. Additionally, a native-like ECM is essential for long-term cell viability and function. Guyette et al. developed native cardiac ECM scaffolds that preserve matrix composition and ultrastructure, supporting the seeding and engraftment of hiPSC-derived cardiomyocytes and enabling the biofabrication of functional human myocardial-like tissue with varying complexity. As conceptually illustrated in Figure 1I, these constructs may offer functional support and ultimately influence heart failure treatment.^107^

Evaluating the therapeutic efficacy and safety of cardiac patches under current Good Manufacturing Practice guidelines is also critical for translation.^108^ Therefore, biomaterial selection is paramount. Recently, both natural biomaterials, such as collagen, alginate, fibrin, and native ECM, and synthetic biomaterials, including polyethylene glycol, poly-ε-caprolactone, and poly(glycerol sebacate), have been utilized in 3D-bioprinted cardiac patches.^109^ Building on preclinical testing, the first clinical trial assessing the safety and efficacy of implanting an iPSC-CM-derived engineered heart patch in patients with end-stage heart failure with reduced ejection fraction is currently underway (the BioVAT-HF study, ClinicalTrials.gov identifier: NCT04396899).^30^

### Conclusions, challenges, and prospects

**Figure 2:**
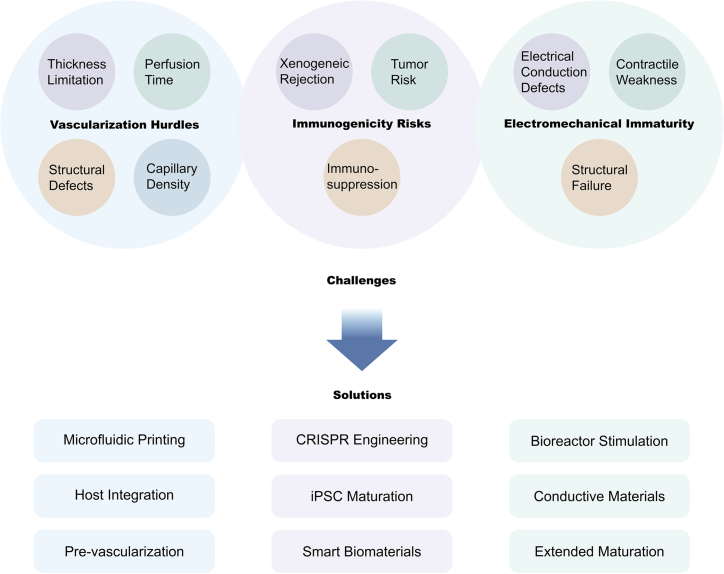
The key challenges in the application of bioprinting and cardiac tissue engineering in cardiovascular diseases

Recent reviews have summarized advances in 3D bioprinting of cardiac tissues using stem cell-derived cardiomyocytes, advanced bioinks, and printing strategies for creating vascularized constructs.^110^^,^^111^ However, a systematic, disease-centered analysis of bioprinting applications across major cardiac pathologies is still needed. Current evidence indicates that traditional CVD treatments, including pharmacotherapy, coronary stent implantation, coronary artery bypass grafting, and organ transplantation, face considerable limitations. Over the past decade, substantial progress has been made toward developing mature bioprinted cardiac tissues through innovations in cell combinations, scaffold design, and culture conditions that incorporate exogenous cues such as mechanical and electrical stimulation.^112^ Despite these advances, bioprinted products remain far from routine clinical implementation. Major challenges persist in applying bioprinting and cardiac tissue engineering to CVDs, including the achievement of functional and scalable vascularization, management of immunogenicity, and attainment of electromechanical maturity in engineered tissues (Figure 2).

The application of 3D bioprinting in MI, cardiac fibrosis, and heart failure reveals distinct therapeutic priorities shaped by their underlying pathophysiology. For MI, the focus is on acute repair and regeneration; bioprinted models are designed to mimic the ischemic border zone, while therapies such as injectable hydrogels aim to rapidly deliver cells and bioactive factors to limit scar expansion and promote angiogenesis. In contrast, research on cardiac fibrosis centers on replicating the chronically stiffened ECM to establish platforms for anti-fibrotic drug screening, though a truly representative 3D model of fibrosis remains an unmet need. For heart failure, the objective is most ambitious: large-scale tissue replacement and remuscularization of the failing ventricle, a goal now being explored in early-stage clinical trials. Despite these differing emphases, the field is unified by the shared platform of the 3D-bioprinted cardiac patch, which commonly utilizes cell sources such as hiPSC-CMs and faces universal translational challenges related to scalability, functional integration, and long-term stability.

The current materials landscape for cardiac 3D bioprinting reflects a strategic multi-material approach, integrating natural biomaterials (e.g., alginate, GelMA, dECM, etc.) for bioactivity,^27^^,^^54^^,^^57^^,^^113^ synthetic polymers (e.g., PCL, PLA, and poly[ester amide]s, etc.) for structural support,^114^^,^^115^^,^^116^^,^^117^ and functional composites (e.g., nanoparticle-loaded systems) for enhanced conductivity and imaging capability^67^ (Table 2). This toolkit is further expanded by scaffold-free strategies such as multicellular spheroids and advanced structural designs like auxetic patches,^53^^,^^68^^,^^87^ collectively addressing the mechanical, electrical, and biological requirements of cardiac tissues. The variety of crosslinking mechanisms, including ionic, photo-, thermal, and enzymatic methods, enables precise control over material properties and printability.^50^^,^^56^^,^^57^^,^^67^^,^^118^ Together, these advances highlight a clear trend toward creating integrated, biomimetic, and patient-specific constructs capable of supporting cardiac regeneration. However, the development of specific constructs, such as heart-valve scaffolds, faces distinct challenges, including the optimization of porosity and interconnectivity, prevention of thrombosis, management of leaflet retraction, and mitigation of calcification. Future progress in materials science will likely focus on dynamic, responsive material systems for *in situ* monitoring and treatment, while addressing these disease-specific design requirements. We propose several directions to address key challenges. First, creating large-scale cardiac tissue requires overcoming the hurdle of functional vascularization. While 3D bioprinting offers superior spatial control and scalability for fabricating vascular networks compared to traditional methods, challenges remain in formulating bioinks and refining fabrication processes to produce vessels with native-like properties and to establish macroscale functional connections within hours after implantation to ensure cardiomyocyte survival.^122^^,^^123^ In 2016, Kolesky et al. developed a vascularized tissue construct that surpassed the conventional 1-cm thickness limit.^124^ Beyond pre-vascularizing bioprinted cardiac tissues, an alternative strategy involves directly connecting host vasculature to engineered constructs.^118^^,^^125^ Although promising, achieving functional macroscale vessel integration remains a significant obstacle, even as microfluidic bioprinting has improved capillary density.^65^ Second, immune responses triggered by xenogeneic materials pose another concern. Researchers are actively exploring hiPSCs and CRISPR-edited immunomodulatory bioinks to mitigate foreign-body reactions.^73^ Current differentiation protocols often yield immature cells; future work must obtain mature iPSCs suitable for bioprinting. Additionally, the time-consuming and costly process of obtaining sufficient high-quality cells limits broader application, and the risk of tumorigenicity requires careful consideration. While immunosuppression targeting T and B cells represents a potential strategy to modulate host responses to allogeneic cells, long-term adverse effects make this approach less desirable.^112^ Otsuka et al. have provided novel insights into current methods for modulating immune responses to transplanted iPSCs.^126^ Third, the electromechanical maturity of engineered tissues frequently falls short of native myocardium, with immature calcium handling and contractile properties being common limitations. This often necessitates extended *in vitro* maturation periods to enhance the functionality of bioprinted cardiac tissues. Overcoming these challenges will be critical for the widespread clinical translation of 3D bioprinting in CVD treatment. Future research should prioritize the optimization of vascularization strategies, improvement of immune compatibility, and enhancement of the electromechanical properties of engineered tissues to bring 3D bioprinting closer to clinical reality.

**Table 2:** Functional materials and mechanisms for 3D bioprinting in cardiac repair

Category	Representative	Mechanism	Advantages	Cardiac applications
Natural biomaterials	Alginate	Ionic crosslinking (Ca^2+^)	Biocompatible, tunable rheology	Heart valves^57^
	Gelatin methacryloyl (GelMA)	Photocrosslinking	Contains RGD sequences, excellent cell adhesion	Disease modeling, cardiac patches^27^^,^^113^
	Fibrin	Enzymatic (thrombin)	Superior cell adhesion and migration	Vascular networks, cardiac tissue^118^
	Collagen	Physical/chemical	Main component of natural ECM	Cardiac patches, NVOT repair^56^
	Hyaluronic acid	Chemical crosslinking	Highly hydrophilic, cell signaling	Valve conduits^57^
	Decellularized ECM (dECM)	Thermal/enzymatic	Patient-specific, preserves native cues	Personalized vascularized patches^54^
	Silk fibroin	Physical/chemical	High strength, controllable degradation	Cardiac patches, vascular grafts^56^^,^^119^
	Chitosan	Ionic/chemical	Antibacterial, cationic properties	Vascular conduits^120^
	GelMA + laminin-521	Photocrosslinking	Supports iPSC-cardiomyocyte growth	Congenital heart disease models^51^
Synthetic polymers	Polyethylene glycol (PEG)	Chemical crosslinking	Tunable properties	Vascular networks, tissue scaffolds^25^
	Polycaprolactone (PCL)	Thermal (thermoplastic)	High mechanical strength, slow degradation	Structural support, vascular stents^114^
	Polylactic acid (PLA)	Thermal (thermoplastic)	Biodegradable, good mechanical properties	Absorbable stents^115^
	Pluronic F127	Thermal (reverse thermal)	Sacrificial material, perfusable channels	Microchannel networks^121^
Composite/nanomaterials	GelMA + gold nanoparticles	Photocrosslinking	Radio-opacity for CT monitoring	Injectable cardiac patches^67^
	PCL + carbon nanotubes	Thermal/physical	Enhanced conductivity and strength	Electrically conductive scaffolds
	GelMA + tantalum nanoparticles	Photocrosslinking	X-ray visibility	Cardiac patches for imaging
Scaffold-free approaches	Multicellular spheroids	Self-assembly	High cell density, native ECM production	Cardiac fibrosis^87^
	Atrioventricular cardiomyocytes	Self-organization	Functionally specialized	VSD repair, electrical conduction^53^
Advanced designs	Auxetic patches	Structural design	Negative Poisson’s ratio, mechanical matching	MI treatment^68^
Bioactive factors	PSTCK1-loaded patches	Sustained release	Angiogenic induction	Vascular regeneration^66^

In conclusion, although still in its early stages and facing significant challenges, 3D bioprinting has achieved substantial progress in cardiovascular regeneration over recent years. Future research should focus on advancing more precise bioprinting techniques, optimizing biomimetic materials, and improving the survival and functionality of bioprinted tissues. As technology evolves and research continues, 3D bioprinting holds promise as an effective therapeutic strategy for CVDs, offering renewed hope for patients.

## Acknowledgments

This work was supported by grants from the 10.13039/501100001809National Natural Science Foundation of China (grant no. 82302085, 82302971), the 10.13039/501100003819Hubei Provincial Natural Science Foundation of China (no. 2024AFD449, 2023AFB190, 2025AFD691), and the Non-profit 10.13039/501100022599Central Research Institute Fund of 10.13039/501100005150Chinese Academy of Medical Sciences (grant no. 2023-PT320-07).

## Author contributions

B.W., J.Z., Y.Z., and C.C. performed the work and wrote the manuscript. C.C., J.J., and Y.Z. planned the research, revised the manuscript, directed, and supervised the study. All authors have read and agreed to the published version of the manuscript.

## Declaration of interests

All authors have no conflicts of interest to declare.
